# Supplementary material for: Cortical direction selectivity increases from the input to the output layers of visual cortex
Source: PLoS Biol. 2025 Jan 8;23(1):e3002947. doi: 10.1371/journal.pbio.3002947 (PMC11709279; doi:10.1371/journal.pbio.3002947)
Supplement: Attachment — Submitted filename: DSLayerCats-ResponsesToReviewers-V10.pdf [file pbio.3002947.s014.pdf]

Dear reviewers,

We would like to express our sincerest gratitude for the time and effort you have dedicated to reviewing our manuscript. We greatly appreciate your recognition of our work and the insightful comments provided.

Below we provide point-by-point responses to your comments. We have underlined your original comments and provided our responses directly beneath, each starting with "Reply" highlighted in bold, red font. Additionally, we have marked all the changes in the revised manuscript with a distinctive yellow highlight for easy identification.

Sincerely,

Dajun Xing

Reviewer #1:
The basic orientation and direction selectivity of V1 neurons have always been one of the major focuses in vision science. In rodents the direction selectivity is initially generated in their retina. In primates and cats, the direction selectivity is firstly emerged in their V1, however, at least two major questions remain elusive regarding direction signal processing. One is the direction signal processing and transmission across hierarchical organized visual pathway such as from V1 to MT and MST in primates, and the other is the laminar processing across input and output layers within a direction selective column particularly in cat V1 and primate MT as these two areas in different species have clearly column organizations of direction selectivity.

By taking advantages of laminar recordings with a multielectrode linear arrays (U-Probe), Dai et. al. studied direction selectivity between input and output layers precisely within a direction-selective column in cat V1. They revealed quantitively the differences of both neuronal response latency and direction selectivity across input and output layers within a single cortical column. They further constructed computational models to reveal a nonlinearity of processing explains the later but higher direction selective responses for the output layer in the preferred direction of a single cortical column. The Granger causality analysis supported a gain amplification of preferred direction selectivity caused by this nonlinearity, which most likely resulted from a combination of feedforward connections from input layer to output layer and recurrent connections within the output layer.

Overall, this is an important and high-quality work, for the first time, Dai et. al. clearly demonstrated differential laminar processing mechanisms for direction selectivity across the input and output layers within a single cortical column in terms of processing timing and direction selectivity strength. Furthermore, the finding of stronger direction selectivity in the output layer is highly consistent with the fact that direction selectivity maps along with orientation maps are predominant features in cat V1, famously revealed by intrinsic signal optical imaging of the output layers of cat V1 something 30 years ago. These results may provide some general insights for the laminar processing of input and output layers for other non-visual sensory signal selectivity within a single column in different primary sensory cortices across species.

However, I do have some minor issues, and also some suggestions regarding the clarity of the manuscript.

For the title and introduction section

1. Line 1 for the title: the title should be more specific regarding the actual laminar mechanism revealed for direction selectivity in this study. So, it would be clearer if the title to be changed as Laminar gain mechanisms for direction selectivity in the primary visual cortex. The gain modulation mechanisms refer a more linear processing for the input layer and a more nonlinear processing for output layer in cat V1, as far as I understand from the current work.

Reply: Thank you for the suggestion. The interlaminar direction-tuned gain in the manuscript refers to the modulation of gains across different directions as signals are processed from the input layers

to the output layers. This direction-tuned gain, combined with nonlinearity, helps enhance direction selectivity in the output layers. It's highly plausible that neuronal processing in the input layers is more linear compared to the output layers, and further studies are needed to understand the generation of direction selectivity in the input layers. Our manuscript, however, mainly focuses on the interlaminar processing of direction selectivity within the early visual cortices.

We have changed the title to "Laminar Gain Mechanisms for Direction Selectivity in the Early Visual Cortices" to make it more specific to the actual laminar mechanisms underlying direction selectivity and to avoid any confusion for the definition of "cat V1".

2. Line 49: Cat V1 traditionally was often called area 17, but not area 18. So, delete /18.

Reply: Thank you for the suggestion. Indeed, area 17 has been traditionally viewed as the primary visual cortex (V1) for cats, but recent discussions also support a broader definition for cat V1. In the seminal book, "The Cat Primary Visual Cortex," Payne and Peters traced the evolution of thinking on the concept of the cat primary visual cortex. Comparing various aspects of areas 17 and 18, including laminar architectures, major subcortical visual inputs, intrinsic circuitries, receptive field properties, and the termination of major efferent projections, they concluded that "What is not in doubt from our discourse is that both areas 17 and 18 should be included under the umbrella term primary visual cortex." (Payne & Peters, 2002). Following this view, many studies (Almasi et al., 2022; Benucci et al., 2007; Finn & Ferster, 2007; Karube & Kisvárday, 2011; Onat et al., 2011; Sun et al., 2021) treated both areas 17 and 18 as cat V1.

In our study, we would like to include data from both areas to increase the generality and the statistic power for our findings. To avoid any confusion for the definition, we have made the following revisions. 1) Instead of using "cat V1", we now use "cat early cortical cortices" throughout the main text. 2) Our findings regarding the interlaminar processing of direction selectivity are consistent between area 17 and area 18, as shown in **Figure R1** here. We have included it in the supplementary information as **Figure S13 (lines 1738-1758, page 61)** to demonstrate this consistency and to provide comprehensive data supporting our conclusions. 3) We also added a discussion comparing the laminar processing of direction selectivity between area 17 and area 18 **(lines 579-591, page 20)** in the revised manuscript.

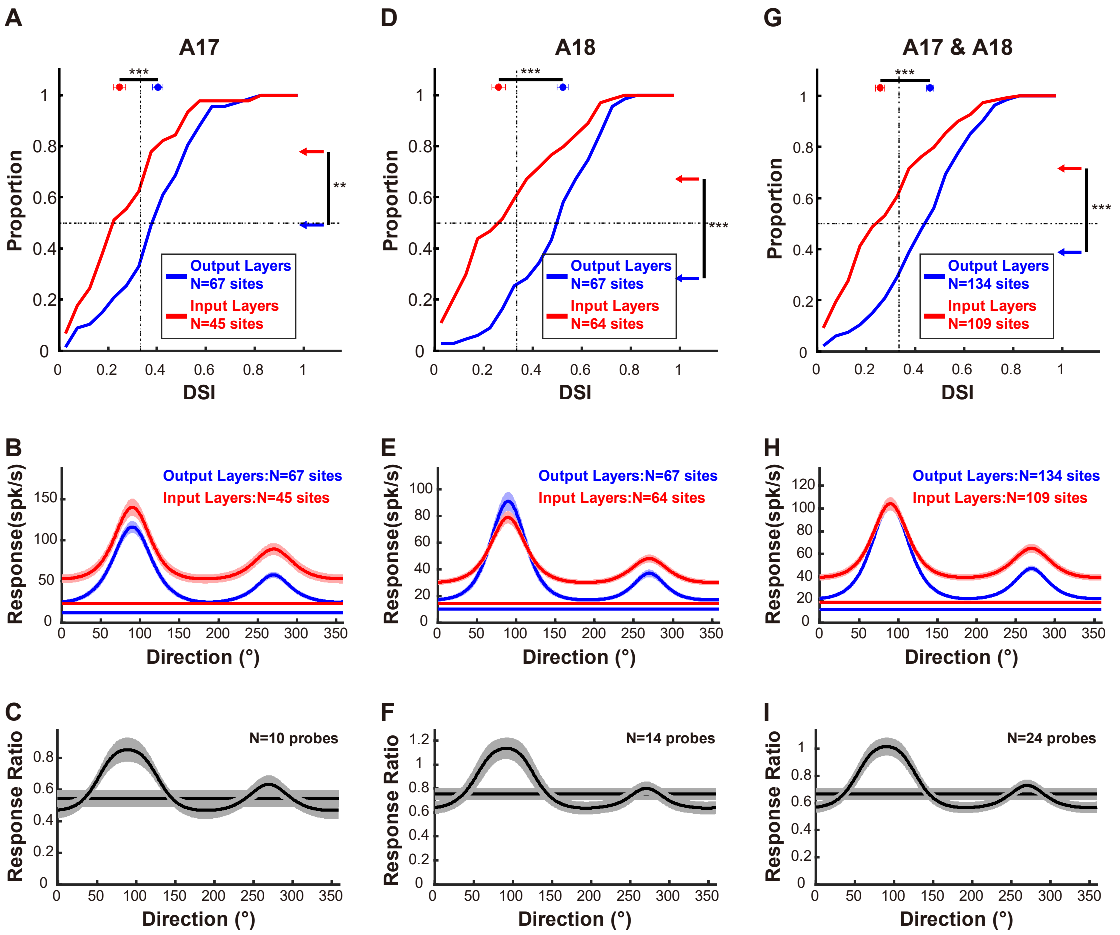

Figure R1. Laminar processing of direction selectivity is consistent across areas 17 and 18.

A, D & G. Cumulative distributions of DSIs in the output layers (blue line) and the input layers (red line). The horizontal dashed line indicates a cumulative proportion of 0.5, and the vertical dashed line represents a DSI of 1/3. The median value and SEM of the DSI for both layers are shown at the top. The proportions of recording sites exhibiting strong direction selectivity (DSI > 1/3) within the input and output layers are denoted by colored arrows on the right. Statistical comparisons for median DSI values (at the top) and proportions of recording sites with strong direction selectivity (on the right) between the output layers and the input layers are also shown.

B, E & H. Population-averaged direction tuning of response strength within the output layers (blue lines) and input layers (red lines). The blue and red horizontal lines represent the average responses to blank stimuli within the output layers and input layers, respectively.

C, F & I. Population-averaged direction tuning of the response ratio between the output layers and the input layers. A-C, results from area 17 (A17); D-F, results from area 18 (A18); G-I, results combining data from A17 and A18.

3. Line 86: "consistent with this hypothesis" seems pointing to the above inheritance from early stages such as LGN for direction selectivity. However, the main sentence talks about the interlaminar connections from other V1 cells for the generation of the selectivity, clearly direction selectivity is not inherited from other V1 direction selective cells. This sentence is confusing and not consistent with the above inheritance hypothesis at all.

Reply: We apologize for any confusion. The inheritance hypothesis suggests that direction selectivity at a given cortical stage in the visual hierarchy is inherited from the previous stage, either

across different cortical areas (e.g., from V1 to MT) or across different layers within a single cortical column (e.g., between the input and output layers of V1). This hypothesis predicts that direction selectivity between two consecutive cortical stages should be correlated, with similar preferred directions. Previous studies have shown that when decomposing the spatiotemporal receptive fields of V1 neurons, the decomposed subunits, considered to be the inputs to the neurons, share similar preferred directions as the neurons. This led us to conclude: "Consistent with this hypothesis, direction-selective cells in V1 are likely to receive synaptic connections from other V1 cells that have similar preferred directions."

We have made the following revisions to clarify any confusion. First, we replaced "later stages" and "earlier stages" with "later cortical stages" and "earlier cortical stages" **(lines 84-85, page 4)**, as is suggested by reviewer #3, to avoid any misunderstanding that direction selectivity in the cortex might be inherited from subcortical regions, especially for carnivores and primates. Secondly, we have included a sentence explicitly describing the prediction of the inheritance hypothesis at **lines 90-92, page 4** in the revised manuscript, ensuring readers understand what part the main sentence is "consistent with".

4. Line 98 - 100. This is talking about the processing of direction signals across different hierarchical visual areas, which is another important question, in addition to the laminar processing mechanism across different cortical layers within a single cortical column. I would like to suggest the sentence of "Therefore, the manner in which direction selectivity is processed and relayed through consecutive stages of the visual hierarchy remains to be fully elucidated", to be changed to a descriptive statement of the past literatures, such as "Therefore, direction selectivity is processed and relayed through consecutive stages of the visual hierarchy".

Reply: Thank you for the suggestion. This paragraph aims to present two contrasting hypotheses on the processing of direction selectivity across consecutive cortical stages: the inheritance hypothesis and the re-establishment hypothesis. These consecutive cortical stages include both different cortical areas and different layers within a single cortical column. To better align with our focus on interlaminar processing of direction selectivity within a single cortical column, we have revised the sentence to: "Given these two contrasting hypotheses, the inheritance hypothesis and the re-establishment hypothesis, the manner in which direction selectivity is processed and relayed through consecutive stages of the visual hierarchy, especially within the neocortex's laminar structure (Douglas & Martin, 2004), remains to be fully elucidated." in the revised manuscript **(lines 103-107, page 4)**.

5. Line 107, replace "within V1 (defined here as Area 17 and 18)" with "within cat area 17 (defined here as V1)", which is more proper and precise. Remove area 18 wherever it appears in the manuscript including figure legends. Cat area 18 is often regarded as the secondary visual cortex, similar to monkey V2.

Reply: Thank you for the suggestion. We have replaced "cat V1" with "cat early visual cortices" throughout the revised manuscript to enhance the generality and statistic power for our findings, as our experimental data showed consistent results between areas 17 and 18. Accordingly, we have

replaced "within V1 (defined here as Area 17 and 18)" with "within the early visual cortices, including areas 17 and 18" on **line 114, page 4**.

6. Line 111, replace "hierarchical" with laminar, which is more precise and proper. Within a cortical area across different processing layers, it is more proper to use laminar, as laminar processing mechanism is the correct and precise term to use here.

Reply: Thank you for the careful reading. We have now replaced "hierarchical" with "laminar" on **line 118, page 5** in the revised manuscript.

7. Line 120, this discovery of gain mechanism could be stated clearly in the title as one sentence summary title.

Reply: We have now changed the title to "Laminar Gain Mechanisms for Direction Selectivity in the Early Visual Cortices".

For the result section

The neuronal response latency provides a vital information for laminar processing across different cortical layers. One advantage of linear recording array across cortical layers is to reveal directly latency differences of distinct processing particularly between input and output layers. Therefore, it would be much better to combine Figure 1AB with Figure S2 as a new Figure 1, to address the distinct latency difference across input and output layers. Further differences for direction selectivity processing across input and output layers shall follow the new Figure 1 as Figure 2, and etc, thereafter.

Reply: Thank you for the suggestion. We have combined Figure 1A-B with part of Figure S2 to create a new **Figure 1** in the revised manuscript **(lines 161-188, pages 7-8)**, as shown in **Figure R2** here. The remaining part of Figure 1 has been moved to the new **Figure 2** in the revised manuscript **(lines 216-235, page 9)**, as shown in **Figure R3** here. The descriptions for the new **Figure 1** and **Figure 2** in the main text have also been adjusted accordingly, at **lines 147-159 on page 6**, and **lines 191-214 on page 8**, respectively.

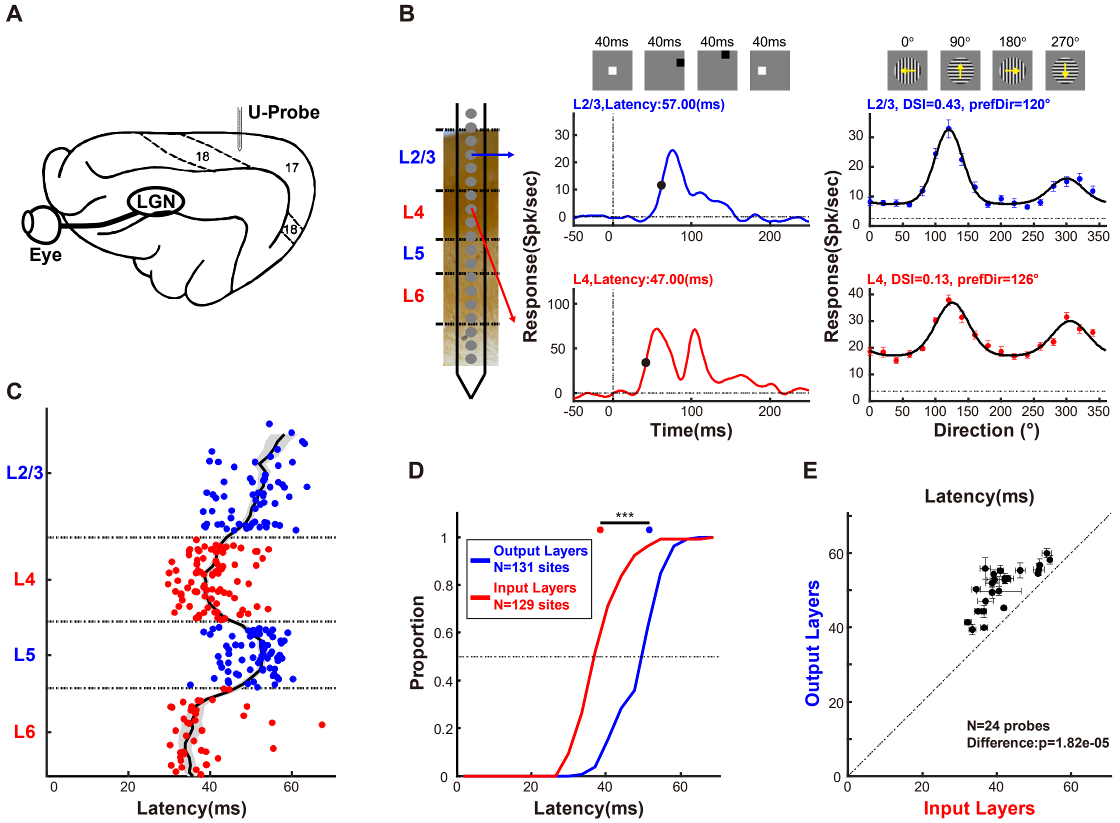

Figure R2. Neuronal responses are delayed in the output layers.

A. Basic methods. We use multi-electrode linear probe to recording laminar responses in cat early visual cortices, including areas 17 and 18.

B. Neuronal responses of example recording sites from L2/3 (upper) and L4 (lower). Left, a linear probe superimposed on a cortical section stained with cytochrome oxidase (CO). Middle, averaged neuronal responses (blue and red lines) to sparse noise (SPN) stimuli located within the receptive field (RF) center. We defined the neuronal response latency (black dots) as the time point at which the response reaches half of its maximum response. The vertical dashed line indicates the stimulus onset time, and the horizontal dashed line represents 0 spk/sec, as the neuronal response was subtracted from the baseline response before stimulus onset. Right, neuronal responses in different directions (blue and red dots), overlaid with direction tuning curves (black solid line) fitted using the von Mises function. The horizontal dashed line indicates the neuronal response to blank stimuli.

C. Laminar pattern of response latency. Each dot represents the data from one recording site. Recording sites belonging to the output layers, including L2/3 and L5, are shown in blue. Recording sites in the input layers, including L4 and L6, are shown in red. The running medians of neighboring recording sites are shown as black lines, and shaded error bars indicate the SEM.

D. Cumulative distributions of response latency in the output layers (blue line) and the input layers (red line). The horizontal dashed line indicates a cumulative proportion of 0.5. The median value and SEM of response latency for input and output layers are shown at the top. Statistical comparison for median response latency between the output layers and the input layers is shown at the top.

E. Comparison of response latency between the output layers and the input layers within the same probe placement. For each probe placement, we obtained the median value and SEM of response latency across all recording sites in the output layers and in the input layers. Thus, each dot represents data from one probe placement. The significance of paired comparisons between the output layers and the input layers is shown.

The error bars are the median ± SEMs; ns: not significant, *$p < 0.05$, **$p < 0.01$, ***$p < 0.001$.

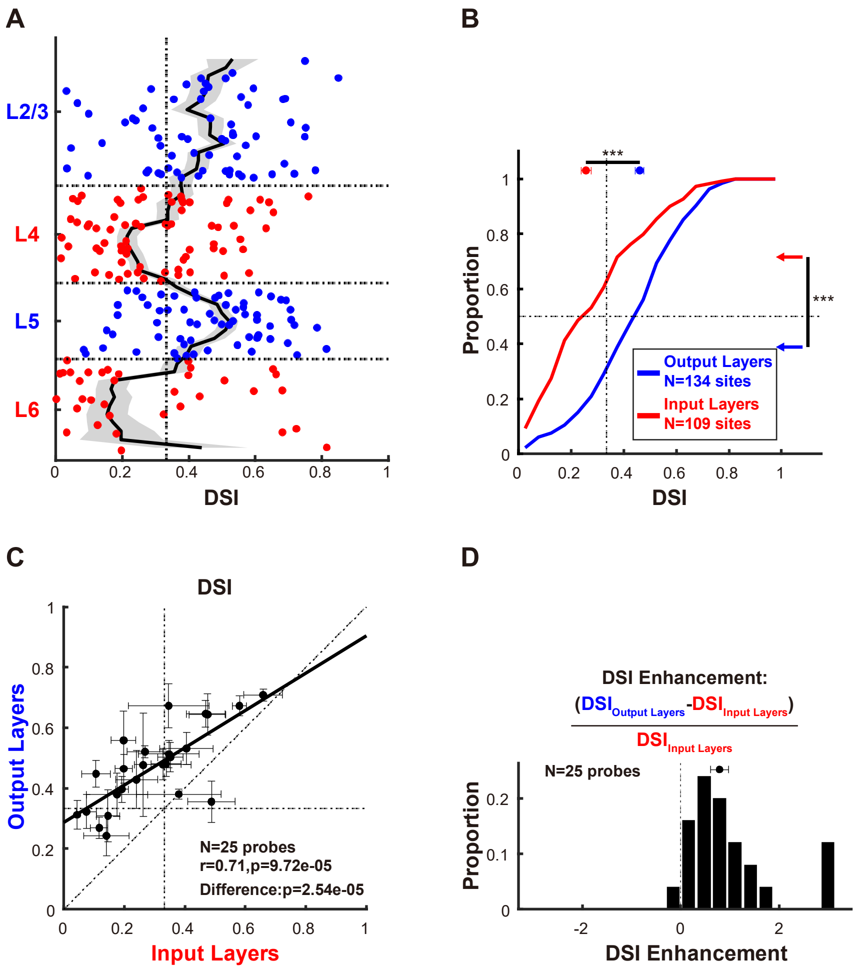

Figure R3. Direction selectivity is enhanced in the output layers.

A. Laminar pattern of the DSI. The vertical dashed line represents a DSI of 1/3, indicating that the response strength in the preferred direction is twice that in the null direction.

B. Cumulative distributions of DSIs in the output layers (blue line) and the input layers (red line). The horizontal dashed line indicates a cumulative proportion of 0.5, and the vertical dashed line represents a DSI of 1/3. The proportions of recording sites exhibiting strong direction selectivity (DSI > 1/3) within the input and output layers are denoted by colored arrows on the right. Statistical comparisons for median DSI values (at the top) and proportions of recording sites with strong direction selectivity (on the right) between the output layers and the input layers are also shown.

C. Comparison of DSIs between the output layers and the input layers within the same probe placement. Thus, each dot represents data from one probe placement. The Spearman correlation and the significance of paired comparisons between the output layers and the input layers are shown.

D. The distribution of DSI enhancements between the output layers and the input layers. The DSI enhancement was calculated as the ratio of the DSI difference between the output layers and the input layers to the DSI in the input layers.

8. Line 281 - 282, The sub-title of "A direction-tuned gain mechanism for interlaminar signal transmission leads to enhanced direction selectivity in the V1 output layer ". The direction-tuned gain mechanism refers to selective response amplification in the preferred direction while less active

in the non-preferred direction. This gain increase for preferred direction in the output layer could well be caused by excitation inputs while the gain decrease (fewer active responses) caused by more inhibitory inputs to non-preferred direction. However, the Granger causality analysis revealed the direction-tuned gain likely stems from a combination of feedforward connections from input layer to the output layer, together with the recurrent connections within the output layer. The authors need to work out or at least to discuss whether the feedforward connections are more likely to be excitatory while the recurrent inputs are inhibitory. Otherwise, the suggestion from Granger causality analysis is less specific.

Reply: Thank you for the suggestion. Based on Granger causality analysis, we found an enhanced feedforward connection from the input layers to the output layers, as well as an increased recurrent connection within the output layers at the preferred direction. The feedforward connection is inherently excitatory according to anatomical studies. However, we cannot determine whether the recurrent connections are due to excitation or inhibition or both. It is possible that stronger recurrent excitation is evoked at the preferred direction, given the presence of direction maps in cat early visual cortices. Previous studies have also reported co-tuned direction tuning of excitatory and inhibitory inputs in the early visual cortices (Li et al., 2015; Monier et al., 2003; Priebe & Ferster, 2005; Znamenskiy et al., 2024), suggesting that stronger recurrent inhibition may also be evoked at the preferred direction. We have added a discussion on the Granger causality results and its implications at **lines 603-608, page 20** in the revised manuscript. Following that, we discussed the potential involvement of excitation and inhibition in shaping direction selectivity and emphasized the importance of studying how the interplay between these two factors contributes to the interlaminar direction-tuned gain.

9. Line 385 (Figure 6C): The scale bar of the heatmap in Figure 6C is inconsistent with that in Figure 6AB, causing the same color in the Figure 6C represents different normalized GC values as in the Figure6AB. It is very hard to compare intuitively and fairly. The higher the normalized GC values, the lower the corresponding p-value. For example, the color of the area representing L4→L5 in the heatmap of Figure 6C shows more yellow than that in the Figure 6A, but the p-value of L4→L5 in the Figure 6C is higher than 0.05 while that in the Figure 6A is lower than 0.001.

Reply: Thank you for the suggestion. We have now set all three panels in this figure to have the same color bar, as shown in **Figure R4** here and the new **Figure 7** in the revised manuscript **(lines 471-481, page 17)**. Additionally, we have also provided paired comparisons of the normalized GC values between different stimulus conditions for selected connection pairs, as shown in **Figure R5** here and **Figure S10** in the revised manuscript **(lines 1697-1704, page 56)**.

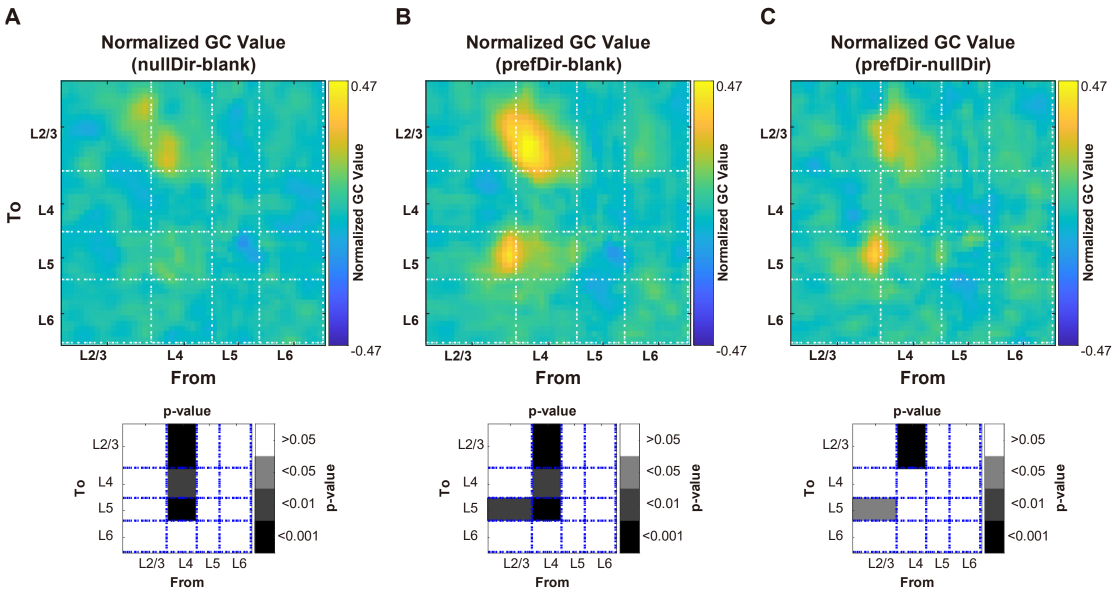

Figure R4. The preferred direction evokes stronger feedforward connections from the input layers to the output layers and recurrent connections within the output layers.

A. Differences in the normalized pairwise-conditional Granger causality (GC) values between the null direction and blank stimulus. The pairwise-conditional GC value for a specific pair of connections is calculated by conditioning out all joint dependencies among the other known variables. This approach helps mitigate the impact of potential confounding factors, such as common inputs. The grayscale inset depicts the p value of the differences in GC values within each connection pair. The source layer (the sender of the projection, from) is positioned along the abscissa, while the target layer (the receiver of the projection, to) is represented along the ordinate.

B. Differences in the normalized GC values between the preferred direction and blank stimulus.

C. Differences in the normalized GC values between the preferred direction and the null direction.

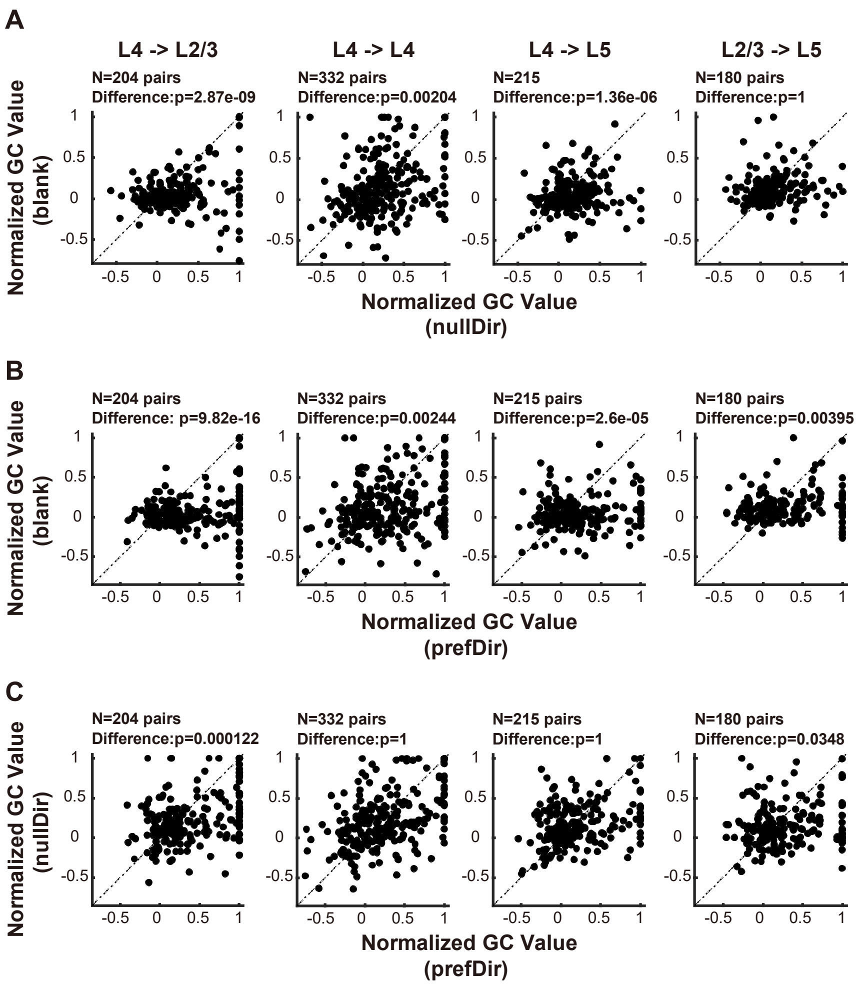

Figure R5. Comparison of normalized GC values between different stimulus conditions.

A. Comparison of normalized GC values between null direction and blank condition. From left to right, GC values are calculated as follows: from L4 to L2/3, from L4 to L4, from L4 to L5, and from L2/3 to L5, respectively. The significance level has been adjusted using the Bonferroni method to account for multiple comparisons, with the p-value shown being multiplied by the number of comparisons (4 * 4 = 16 comparisons).

B. Comparison of normalized GC values between preferred direction and blank condition.

C. Comparison of normalized GC values between preferred direction and null direction.

For the discussion section

<u>10. Line 421: Replace "Hierarchical processing of direction selectivity in V1" with "Laminar processing of direction selectivity in V1". This is because again that Laminar processing is more precise term to use than hierarchical processing in the current study, the later often refers processing across different visual areas rather than different cortical layers. Both Laminar and hierarchical processing are equally important in the visual brain.</u>

Reply: Thank you for the careful reading. We have now changed the sub-title to "Laminar processing of direction selectivity in the early visual cortices" at **line 503, page 18** in the revised manuscript.

11. Line 430 - 433: delete" Whether the strategy observed within a single cortical column of cat V1 is also utilized across two consecutive stages from different cortical areas (for example, from V1 to MT and from V1 to V2) remains an open question for future research." This is a less relevant question to the current study so no need to be commented here. Instead, it would be more relevant talking about there is a direction map in cat V1, but not in monkey V1, so that cat V1 provides an idea model to study laminar processing of direction selectivity across input and output layers (with citation for the direction maps recorded with intrinsic signal optical imaging). It would be more relevant to talk about cat area 18, which often regarded as equivalent to monkey V2 thick stripes.

Reply: Thank you for the suggestion. We have deleted the sentence and added evidences supporting the cat early visual cortices as an appropriate model for studying the laminar processing of direction selectivity **(lines 512-518, page 18)**. Additionally, we have included a discussion comparing the laminar processing of direction selectivity between A17 and A18 **(lines 579-591, page 20)**. Based on this comparison, we suggest that it would be interesting to explore whether interlaminar processing of direction selectivity varies across different cortical regions, such as between monkey V2 thick stripes and MT.

12. Line 515: It is difficult to say whether the use of anesthetized animals is a disadvantage for studying basic visual features such as orientation and direction selectivity. It will be definitely the case for studying any behavior visual task in anesthetized animals.

Reply: We totally agree that using anesthetized animals may not be a disadvantage when studying the cortical processing of basic visual features. We have revised the sentence to: "In the present study, we recorded neuronal responses in anesthetized animals, which is well-suited for studying cortical processing of basic visual features. However, the use of anesthetized animals presents two inherent limitations." at **lines 678-680, page 22**. Following that, we discussed the potential limitations of using anesthetized animals: one being the possible attenuation of feedback connectivity under anesthesia, and the other that it remains unclear how propofol affects neuronal responses across the cortical column.

13. Line 527 - 529: The reviewer does think these are advantages, not the limitation, as it is only fair for a direct comparison of direction selectivity between input and output layers, by using drifting gratings with fixed stimulus properties such as contrast, size, spatial frequency, and temporal frequency, with only the direction varying. Otherwise, it is hard and unfair to do direct comparison if stimuli vary with more parameters.

Reply: We agree that varying only the direction while keeping other stimulus properties constant is both fair and advantageous for directly comparing direction selectivity across different layers. We have revised the sentence to: "By using drifting gratings with fixed stimulus properties such as contrast, size, spatial frequency, and temporal frequency, and by varying only the direction, we can directly compare direction selectivity between the input and output layers through simultaneous recordings." at **lines 692-695, page 22**. Following that, we discussed that since both direction selectivity and laminar connectivity are reported to be modulated by the spatiotemporal configurations of stimuli, it would be a fruitful future direction to study laminar processing and its

underlying mechanisms with a richer array of stimuli.

14. Finally, as simple and complex cells are famously found in the input layer (more simple cells) and the output layer (more complex cells in layer 2/3) in cat V1 more than 50 years ago, how these two cell types may contribute differently to the direction-tuned gain mechanism. This needs to be at least discussed, in addition to excitatory and inhibitory connections, which could link directly with the direction-tuned gain mechanisms (linear and non-linearity).

Reply: Thank you for the suggestion. In **Figure R6A**, we showed the laminar distribution of the modulation ratio (F1/F0), defined as the ratio between the response at the first harmonic (F1) and the averaged response (F0) from sorted single-unit data. It is clear that the input layers exhibit stronger modulation than the output layers **(Figure R6A-B)**. For both simple cell population (F1/F0 $\geq$ 1) and complex cell population (F1/F0 < 1), direction selectivity is increased from the input layers to the output layers **(Figure R6C-D)**. We then analyzed the correlation between F1/F0 and DSI within the output and input layers, and found no correlation in either layer **(Figure R6E-F)**. Furthermore, there exists no significant difference in direction selectivity between simple cells and complex cells within either the output layers or the input layers **(Figure R6G-H)**. These results suggest that the interlaminar processing of direction selectivity is uncorrelated with cell types (simple cells vs complex cells), in line with previous studies in cat early visual cortices (An et al., 2014) and monkey V1 (Lochmann et al., 2013; Rust et al., 2005).

We have included these results as **Figure S11** in the supplementary information at **lines 1707-1722, pages 57-58** and added a discussion on the relationship between DSI and modulation ratio at **lines 553-564, page 19** in the revised manuscript.

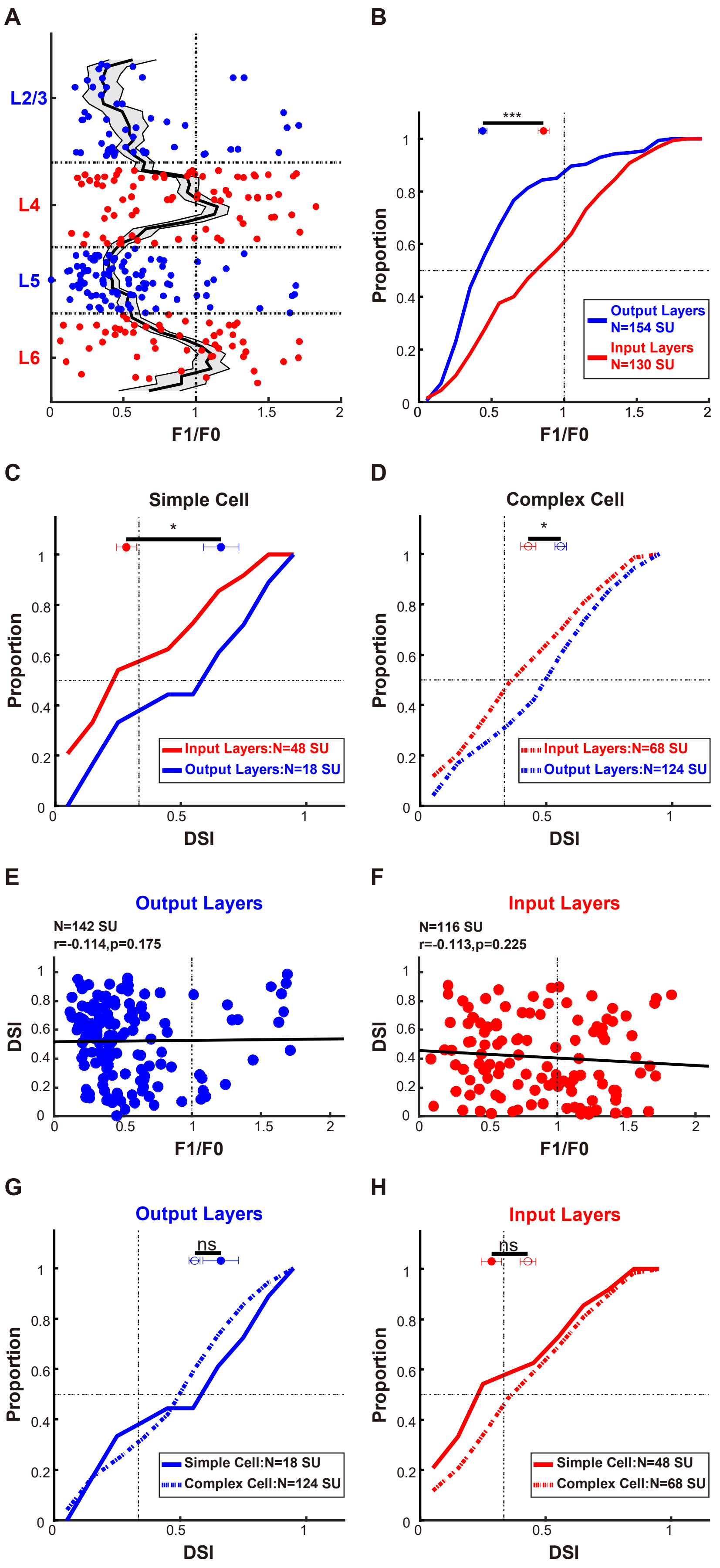
A
B
C
D
E
F
G
H
L2/3
L4
L5
L6
F1/F0
Proportion
***
Output Layers N=154 SU
Input Layers N=130 SU
Simple Cell
Complex Cell
*
Input Layers:N=48 SU
Output Layers:N=18 SU
Input Layers:N=68 SU
Output Layers:N=124 SU
DSI
Output Layers
Input Layers
N=142 SU
r=-0.114,p=0.175
N=116 SU
r=-0.113,p=0.225
ns
Simple Cell:N=18 SU
Complex Cell:N=124 SU
Simple Cell:N=48 SU
Complex Cell:N=68 SU

Figure R6. Direction selectivity strength shows no correlation with the modulation ratio.

A. Laminar pattern of the modulation ratio (F1/F0). The vertical dashed line represents a F1/F0 of 1, indicating that the averaged response (F0) and the modulated response at the stimuli's temporal frequency (F1) are of the same strength.

B. Cumulative distributions of F1/F0 in the output layers (blue line) and the input layers (red line). The horizontal dashed line indicates a cumulative proportion of 0.5, and the vertical dashed line represents a F1/F0 of 1. The modulation ratio is significantly larger in the input layers than in the output layers.

C. Cumulative distributions of DSI for all simple cells ($F1/F0 \geq 1$) in the output layers (blue line) and the input layers (red line). Direction selectivity is significantly increased from the input layers to the output layers for simple cells.

D. Similar to C but for results from complex cells ($F1/F0 < 1$).

E. DSI shows no significant correlation with F1/F0 in the output layers.

F. DSI shows no significant correlation with F1/F0 in the input layers.

G. Cumulative distributions of DSI for all simple cells (solid lines) and complex cells (dashed lines) in the output layers. There exists no significant difference in direction selectivity strength between simple cells and complex cells within the output layers.

H. Similar to G but for results from the input layers.

Reviewer #2:

In this article, Dai and colleagues examined laminar processing of visual information with regard to the direction of stimulus movement in cat primary visual cortex. Using the multielectrode linear array, they recorded the multiunit spiking activity and quantified the strength of directional selectivity across the depth of a cortical column. The authors found that the directional selectivity strengths of input and output layers are correlated, and that directional selectivity is amplified in the output layers compared to the input layers. The authors discuss a possible gain mechanism for the signal transmission from input layers to output layers that leads to an enhanced directional selectivity in the output layers. Using computational modeling, the authors conclude that the amplification derives from the interplay between the feedforward interlaminar connections and the recurrent intralaminar connections.

Apart from a few minor concerns, the results of this study seem solid, the experimental work and the analysis are carefully performed, and the manuscript is well written. I would like to propose the following points for better understanding of the results.

In the abstract, the authors state that they studied "the V1 input layer and the V1 output layer" However, only three pages later, we learn that "input layer" includes layers 4 and 6, and that "output layer" refers to the combined layers 2/3 and 5. It is confusing and might be clearer to pluralize the word "layer" or use the more established term of input/output stage.

Reply: Thank you for the suggestion. We have changed "input layer" and "output layer" to "input layers" and "output layers" throughout the revised manuscript, respectively.

What are the orientation selective indices for the directional selective MUAs identified in this study? I find it interesting that what looks like an orientation column in the input layers becomes a direction column at the later stage of processing. There could be two explanations for the low DSI values. Either the neuronal activity is selectively tuned to the oriented stimuli showing nearly equal responses to the two opposing directions, or it is broadly tuned and responds to all directions. It would be helpful to add a OSI distribution across the cortical depth.

Reply: Figure R7A-B shows the OSI distribution across cortical depth, revealing stronger orientation selectivity in the output layers compared to the input layers, which is consistent with previous studies (cat: (Martinez et al., 2002); monkey: (Ringach et al., 2002; Wang et al., 2020)). We also found a strong positive correlation between DSI and OSI within both the output and input layers (**Figure R7C**), suggesting that the generation of orientation and direction selectivity may share partially overlapping mechanisms, such as intracortical inhibition (Lee et al., 2012; Priebe & Ferster, 2008; Xing et al., 2011) or spike threshold (Priebe, 2016).

Regarding the two possible explanations for the low DSI values that you proposed, we believe our data support the first possibility: neurons are orientation-tuned while exhibiting similar responses to two opposing directions, especially in the output layers. **Figure R7D-I** presents the results for OP ratio (the ratio of responses between the orthogonal orientation and the preferred orientation) and HWHH (half-bandwidth at half-height), two additional measures of orientation selectivity. Most

recording sites in the output layers have an OP ratio below 0.4, though some still exhibit weak direction selectivity. In the input layers, while the orientation tuning is broader compared to the output layers, the OP ratio/HWHH values for most recording sites are below 0.6/60 degrees, indicating that they are still tuned to orientation.

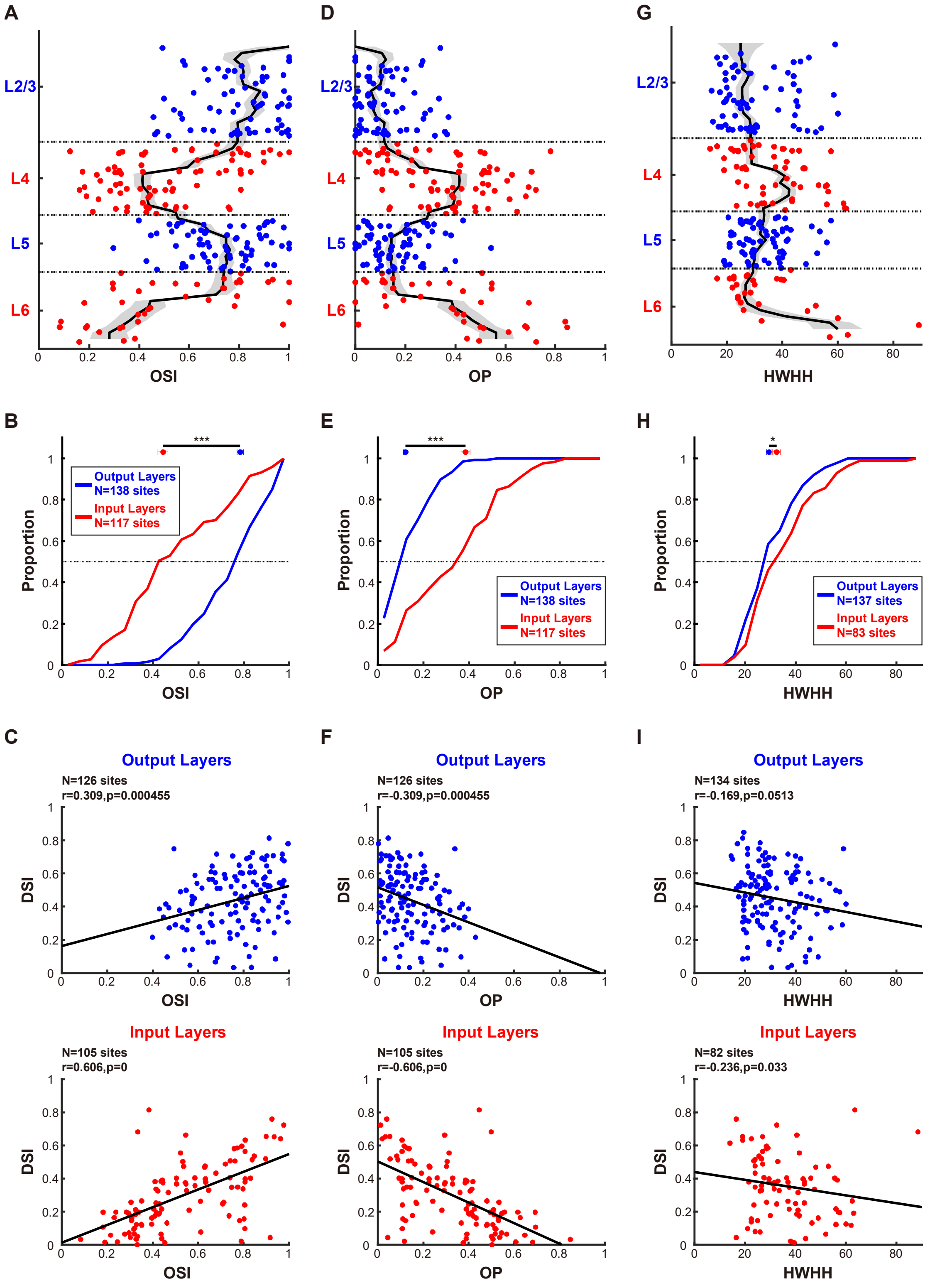

Figure R7. Strong correlation between direction selectivity and orientation selectivity.

A. Laminar pattern of the orientation selectivity index (OSI). The OSI was calculated as $\frac{R(prefOri)-R(orthoOri)}{R(prefOri)+R(orthoOri)}$, where $R(prefOri)$ and $R(orthoOri)$ are the neuronal responses in the preferred orientation and in the orthogonal orientation, respectively.

B. Cumulative distributions of OSI in the output layers (blue line) and the input layers (red line). The orientation selectivity is significantly stronger in the output layers than in the input layers.

C. DSI are strongly correlated with OSI in both the output layers (left) and the input layers (right).

D-F. Similar as A-C, but for the results of OP ratio. The OP ratio was calculated as $\frac{R(orthoOri)}{R(prefOri)}$.

G-I. Similar as A-C, but for the results of HWHH. HWHH is defined as the half-width at half-height of the maximum response.

We have included these results in the supplementary information as **Figure S12 (lines 1725-1735, pages 59-60)** and added a discussion on the relationship between orientation selectivity and direction selectivity and their laminar dependence at **lines 566-577, page 19** in the revised manuscript.

In line 435, the sentence "Based on a database of 899 cells, Kim et al. characterized the distribution of DSI across different layers of cat V1 (Kim & Freeman, 2016)" appears to be an incomplete statement and is not tied to the discussion. It would be interesting to see the authors discuss especially the difference in L6 DSI distribution.

Reply: Thank you for the suggestion. In the work by Kim & Freeman (Kim & Freeman, 2016), which focused on cat A17, they also observed a higher proportion of direction-selective neurons in L2/3 and L5 compared to L4, consistent with our data. However, they found that the DSI distribution peaked at high values in L6, which we did not observe. We discussed that this difference could be due to the complexity of L6 and our smaller sample size in this layer. One study in monkey V1 identified six functional classes in L6, with one highly direction-selective cluster located in the upper half of L6 (Hawken et al., 2020). Additionally, the direction map in cat A17 is patchy rather than homogeneous (Nishiyama et al., 2019). These factors suggest that sampling at different cortical positions or depths may influence the DSI distribution in L6. We have added this discussion at **lines 523-535, pages 18-19** in the revised manuscript.

There was some promise made in the manuscript, and the whole paragraph written in the Methods, about identifying the single unit recordings based on the spike waveform, but these interesting data have not been analyzed and interpreted in the results or the discussion.

Reply: The main purpose of performing single-unit analysis is to demonstrate that, in line with MUA data, we observed stronger direction selectivity in the output layers and a consistent preferred direction between the input and output layers based on single unit data **(Figure R8A-B)**.

We have classified all recorded single units into five classes based on their spike waveforms: narrow-spiking units, broad-spiking units, triphasic-spiking units, compound-spiking units, and positive-spiking units. Of these, four classes (excluding narrow-spiking units) may represent either

excitatory neurons within the early visual cortices or thalamocortical inputs, which should also be considered excitatory (Sun et al., 2021). We showed that even when only considering these putative excitatory neurons within the early visual cortices, we still observed similar results as seen with MUA **(Figure R8C-D)**.

We then characterized the interlaminar comparison of direction selectivity for narrow-spiking units. We found no significant difference in direction selectivity between the input and output layers, as direction selectivity was already strong in the input layers **(Figure R8E-F)**. However, it is uncertain whether this finding can be attributed to the typical response properties of inhibitory neurons in cat early visual cortices, as: 1) the sample size of narrow-spiking units in the input layers is relatively small (28 units), and 2) classification of neuronal types based on spike waveform remains controversial. Recent studies have shown that some narrow-spiking units in cat early visual cortices and monkey V1/premotor/motor cortex may belong to excitatory neurons (Gray & McCormick, 1996; Onorato et al., 2020; Vigneswaran et al., 2011), while some GABAergic neurons in mouse V1 exhibit broad-spiking waveforms (Gentet et al., 2010). Moreover, results on the direction selectivity of inhibitory neurons are diverse (cat early visual cortices (Azouz et al., 1997; Nowak et al., 2008); ferret V1 (Chang & Fitzpatrick, 2022)), highlighting the need for future studies to characterize the response properties of inhibitory neurons within the early visual cortices and their functional roles within neocortical networks.

We have included these results in the supplementary information as **Figure S4 (lines 1603-1631, pages 49-50)** and added a discussion at **lines 644-659, page 21** in the revised manuscript.

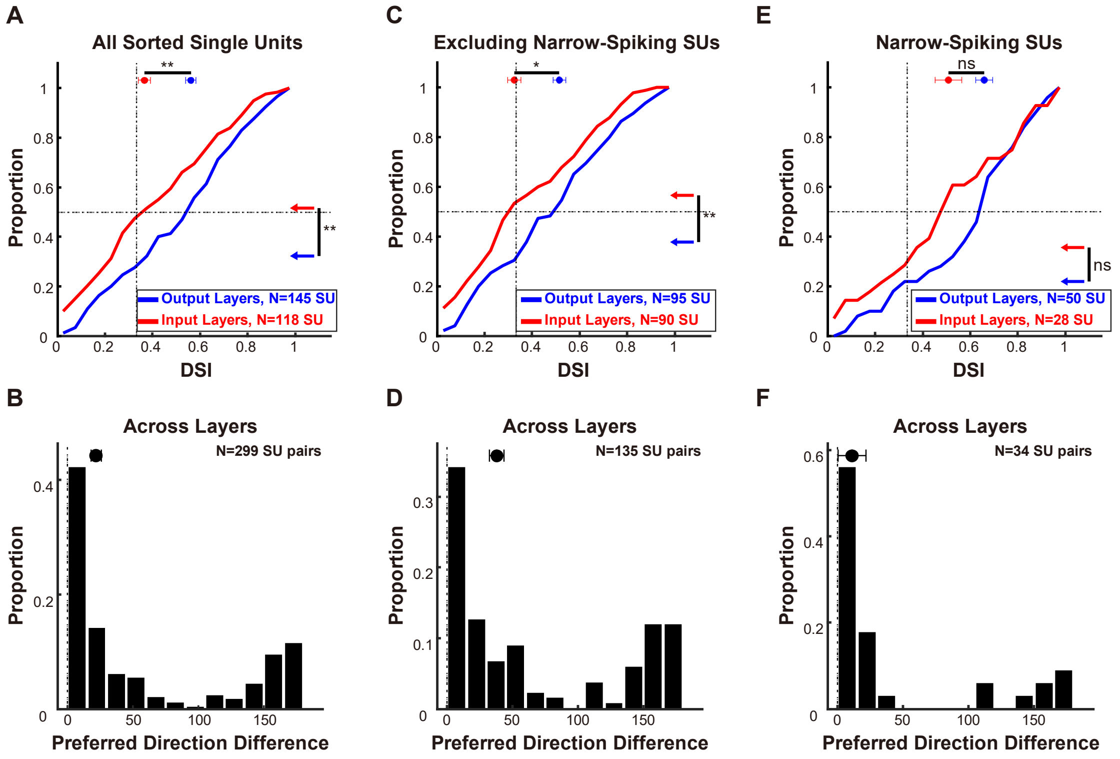

Figure R8. Similar results to MUA when considering only putative excitatory neurons.

A. Cumulative distributions of DSI in the output layers (blue line) and input layers (red line) for all sorted single units. Direction selectivity is significantly stronger in the output layers than in the input layers.
B. Distribution of differences in preferred direction for neuronal pairs between input and output layers.
C-D. Similar to A-B, but for single units excluding narrow-spiking units.
E-F. Similar to A-B, but for narrow-spiking units only.

Lines 696-700 in Figure S6A-E should be S3A-E.

Reply: Thank you for your careful reading. We have now revised it at **lines 870-875, page 27**.

In Figure S3B, the caption inside the box should be L2/3 instead of L23.

Reply: Thank you for your careful reading. We have now revised it at **line 1570, page 47**.

Reviewer #3 (Jose-Manuel Alonso):

This is an interesting paper that advances our understanding of how direction of motion is processed in primary visual cortex. The authors performed careful simultaneous measurements of direction selectivity at different layers of the cat visual cortex with multielectrode probes and, by doing so, they provide the first strong evidence that cortical direction selectivity increases from the input to the output layers of visual cortex. The data is of high quality and the authors convincingly demonstrate that their probes were carefully aligned with the perpendicular axis of the cortex (i.e. they recorded from neurons sharing the same retinotopy, orientation, and direction preference), which is an important requirement to obtain accurate input/output comparisons. The comments below are all relatively minor.

Main comments

1) The authors motivate the study by saying that 'It is believed that direction selectivity in later stages substantially inherits characteristics from earlier stages'. This statement can be very confusing to the reader because the terms 'later' and 'earlier stages' are too broad. For example, if 'earlier stages' refers to retina, the authors would be ignoring an extensive literature in carnivores and primates (starting with Hubel and Wiesel), which supports a cortical origin of direction selectivity. Whereas both cats and primates are likely to have a small subpopulation of retinal ganglion cells that are direction selective, few would argue that cortical direction selectivity is inherited from this small subpopulation in carnivores and primates. Consider adding the term 'cortical' after 'later' and 'earlier' in this sentence ('It is believed that direction selectivity in later cortical stages substantially inherits characteristics from earlier cortical stages'). Without 'cortical', this sentence implies that direction selectivity in visual cortex is inherited from direction selectivity in retina, which is not what was proposed by Adelson & Bergen, 1985 and many other studies in carnivores and primates. The authors can still argue that some cortical direction selectivity in rodents and lagomorphs is likely to be inherited from the retina, but there is no evidence suggesting that this is also the case for carnivores and primates (e.g. direction selective retinal ganglion cells in carnivores and primates could be part of the Koniocellular pathway that projects directly from the thalamus to area MT and not V1).

Reply: We apologize for the confusion and thank you for the suggestion. We agree that the inheritance hypothesis of direction selectivity for carnivores and primates applies only to consecutive cortical stages. Following your suggestion, we have made the corresponding revisions (adding the term "cortical" after "later" and "earlier" in the sentence) at **lines 84-85, page 4** in the revised manuscript and we also adjusted the terms throughout the main text.

2) A main conclusion from the paper is that responses to the preferred direction are selectively amplified from the input to the output layers of the cortex. This conclusion is consistent with model II, but there are other alternative conclusions/interpretations. For example, based on the average illustrated in Figure 3A, it could be argued that the response to the preferred direction is preserved from input to output layers of V1, but the responses to all other non-preferred directions are selectively suppressed. Alternatively, and based on the distribution of response ratio for preferred direction illustrated in Figure 3C-D, it could be argued that the response to the preferred direction

is enhanced in some cortical columns but not others, whereas the response to the blank is suppressed in nearly all cortical columns measured. Consistent with this argument, the output layers could receive global suppression for all directions and selective enhancement for the preferred direction. The authors may want to simulate some of these mechanisms to investigate whether their model II can be more accurate if it incorporates both intracortical excitation and inhibition.

Reply: Thank you for the suggestion. In our Model II, we assumed that neuronal responses in the input layers are first multiplied by a tuned gain and then processed through a nonlinearity to simulate responses in the output layers. The nonlinearity we used is a threshold-linear transfer function, which simulates the transformation of instantaneous voltage to firing rate and assumes Gaussian noise in the voltage. This form of nonlinearity, along with the expansive power-law nonlinearity (results shown in **Figure S8**), both implicitly/potentially include a threshold-like suppressive component.

To explicitly incorporate both intracortical excitation and inhibition, we simulated two additional models corresponding to the alternative possibilities you proposed: one with tuned gain and untuned suppression (Model III) and the other one with untuned gain and tuned suppression (Model IV). In both Model III and Model IV, input layer responses are first multiplied by a linear gain, then subtracted by a suppressive component, and finally processed through a rectified-linear nonlinearity (ReLU) to simulate output layer responses **(Figure R9A)**.

We found that Model II outperforms Model III (tuned gain and untuned suppression) by a very small margin **(Figure R9B)** and shows similar performance as Model IV (untuned gain and tuned suppression, **Figure R9C**). Based on Model III, we also found a direction-tuned gain **(Figure R9D)**, the direction selectivity of which highly correlated with the DSI of the output layers **(Figure R9E)**. These results closely resemble those from Model II.

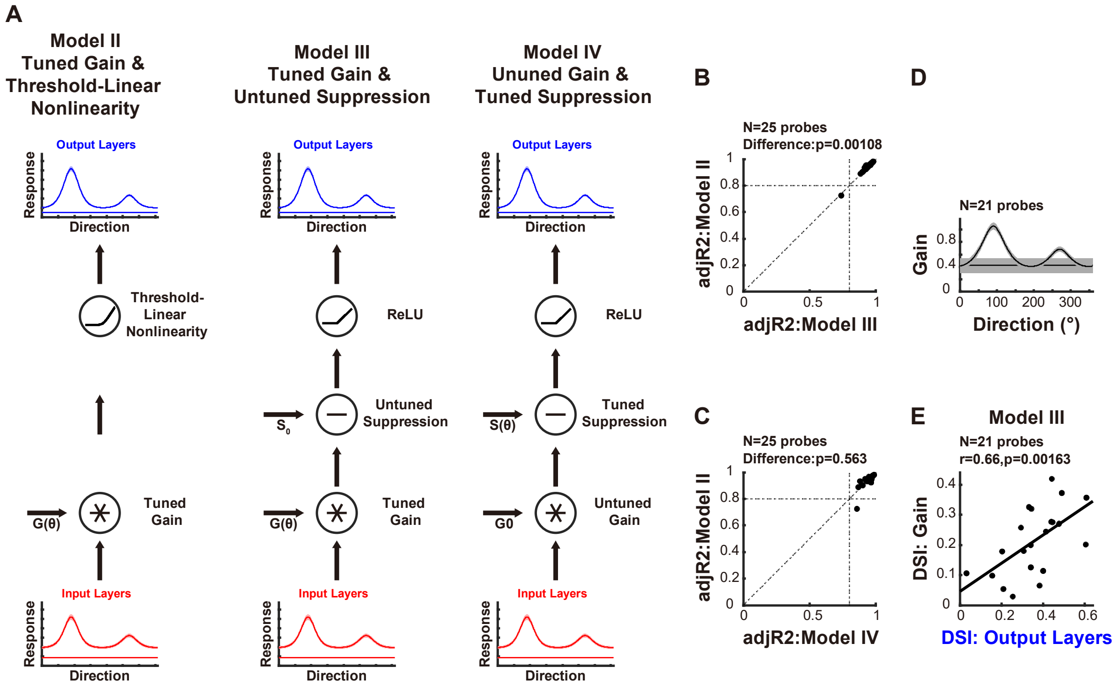

Figure R9. Similar performance as Model II for models with a gain-suppression cascade.

A. Model structure. Left, Model II in the main text. Middle & Right, neuronal responses in the input layers are first multiplied by a linear gain (G), then subtracted by a suppressive component (S), and finally passed through a rectified-linear nonlinearity (ReLU) to simulate output layer responses. In Model III (middle panel), the linear gain is direction-tuned while the suppression is untuned to direction. In Model IV (right panel), the linear gain is untuned to direction and the suppression is direction-tuned.

B. Comparison of adjusted fitting goodness ($adjR^2$) across Model II and Model III. Model II outperforms Model III by a very small margin ($adjR^2$ of Model II VS Model III: 0.9502 ± 0.0101 VS 0.9429 ± 0.0096).

C. Comparison of adjusted fitting goodness ($adjR^2$) across Model II and Model IV. Model II and Model IV shows no significant difference ($adjR^2$ of Model II VS Model IV: 0.9502 ± 0.0101 VS 0.9492 ± 0.0073).

D. Population-averaged direction tuning of the gain in Model III.

E. The DSI of gain in Model III highly correlates with that of the output layers.

Given the similar performance of these models, our data are not sufficient to dissociate excitatory and inhibitory contributions to laminar processing of direction selectivity. However, our results highlight the importance of nonlinearity and direction-tuned gain, which may involve a combination of both excitation and inhibition, in the interlaminar processing of direction selectivity. As noted in the Discussion, future studies could directly manipulate layer-specific excitatory or inhibitory neurons to investigate their corresponding roles.

We have included these model results in the supplementary information as **Figure S9** at **lines 1681-1694, page 55** of the revised manuscript. Additionally, we presented results from Model III in the Results section at **lines 445-453, page 16**, and discussed results from Model IV in the Discussion section at **lines 663-665, page 21**.

<u>3) The authors may want to report the correlation coefficients and correlation significance in the three panels of Figure 3D. There is clearly no correlation between response ratios to blank and null direction. However, there may be a significant correlation between response ratios to blank and preferred direction and there is definitely a strong correlation between response ratios to null and preferred direction. Also, by challenging the simulations to replicate these response-ratio correlations, the authors may make the model more realistic. For example, Figure 5C demonstrates that the model can replicate some of the measurements from Figure 3D. However, Figure 5C appears to reveal a strong correlation between the ratios of blank and nullDir that does not match the measurements from Figure 3D. Is this because they are plotting the gain of the ratios instead of the stimulated output responses (gain + nonlinearity) or is it because they are not using global suppression separately from gain? Again, testing a few variations of model II may help to identify the one that best matches the measurements from Figure 3D.</u>

Reply: Thank you for the suggestion. We have added the correlation coefficient and correlation significance in the three panels of the new **Figure 4D** (shown here as **Figure R10A**). As you correctly observed, the relationships between response ratios under different stimulus conditions vary: there is no correlation between the null direction and the blank condition, a negative correlation between the preferred direction and the blank condition, and a positive correlation between the preferred and null directions.

The difference between Figure 5C (**Figure 6C** in the revised manuscript) and Figure 3D (**Figure 4D** in the revised manuscript) is that Figure 5C presents comparisons of gains from Model II, while Figure 3D shows comparisons of response ratios in the data. **Figure R10B** displays the response ratio between the simulated output layer responses in Model II and the input layer responses. It is clear that Model II replicates the relationship of response ratios across different stimulus conditions very well. We have included a description at **lines 307-311, pages 11-12** and added these results to the supplementary information as **Figure S7 (lines 1657-1664, page 53)** in the revised manuscript.

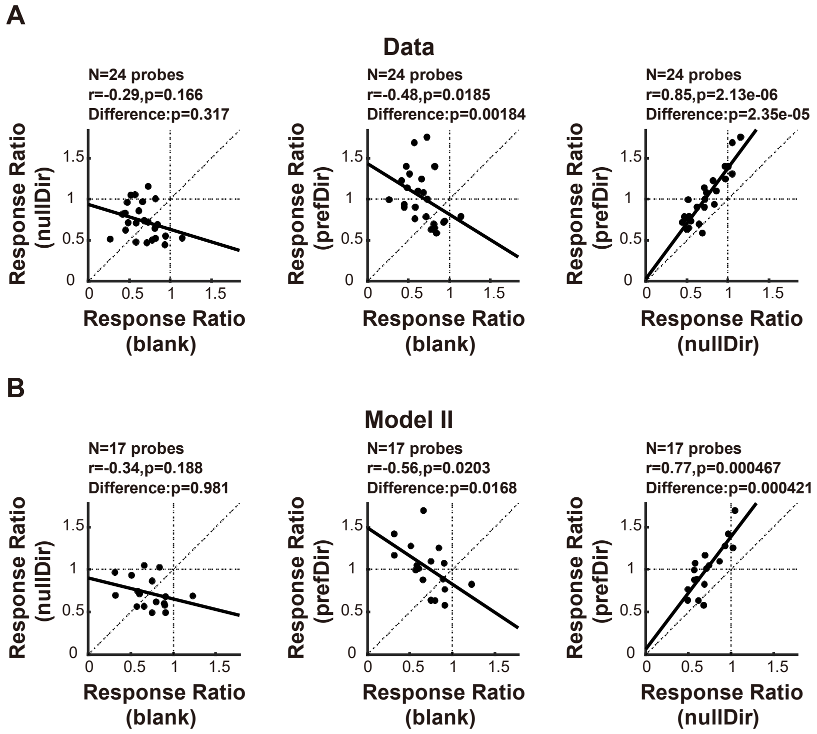

Figure R10. Model II accurately replicates the relationships of response ratios between different stimulus conditions as observed in the data.

A. Relationship of response ratios between different stimulus conditions in the data. There is no correlation between the null direction and the blank condition (left), a negative correlation between the preferred direction and the blank condition (middle), and a positive correlation between the preferred and null directions (right).

B. Similar to A, but for results from Model II.

4) The classification of recordings into input and output layers is a reasonable approach to increase the statistical power of the dataset. However, if the authors have enough statistical power, it would be helpful to replicate Figure 3 in a supplementary figure just for comparisons between layer 4 and layers 2+3. This comparison is particularly important because layer 4 provides strong monosynaptic input to layers 2+3. However, the connections linking other input and output layers are much weaker (e.g. layer 6 to layer 2+3 or layer 4 to layer 5). The authors can ignore this comment if the sample size is not enough to restrict the comparisons to layer 4 and layers 2+3.

Reply: We have replicated Figure 3 as part of the new **Figure S3 (lines 1570-1600, pages 47-48)** to restrict the comparisons to L4 and L2/3, shown here as **Figure R11 (Figure R11D-G)**. The results

between L4 and L2/3 are nearly identical to those between input and output layers, except that the median response ratio between L2/3 and L4 at the preferred direction is slightly less than 1, whereas the ratio between output and input layers is not significantly different from 1. This difference does not affect our main conclusion, as direction selectivity strength is still enhanced from L4 to L2/3 **(Figure R11A-C)**, and an interlaminar direction-tuned gain from L4 to L2/3 is also present **(Figure R11H-I)**.

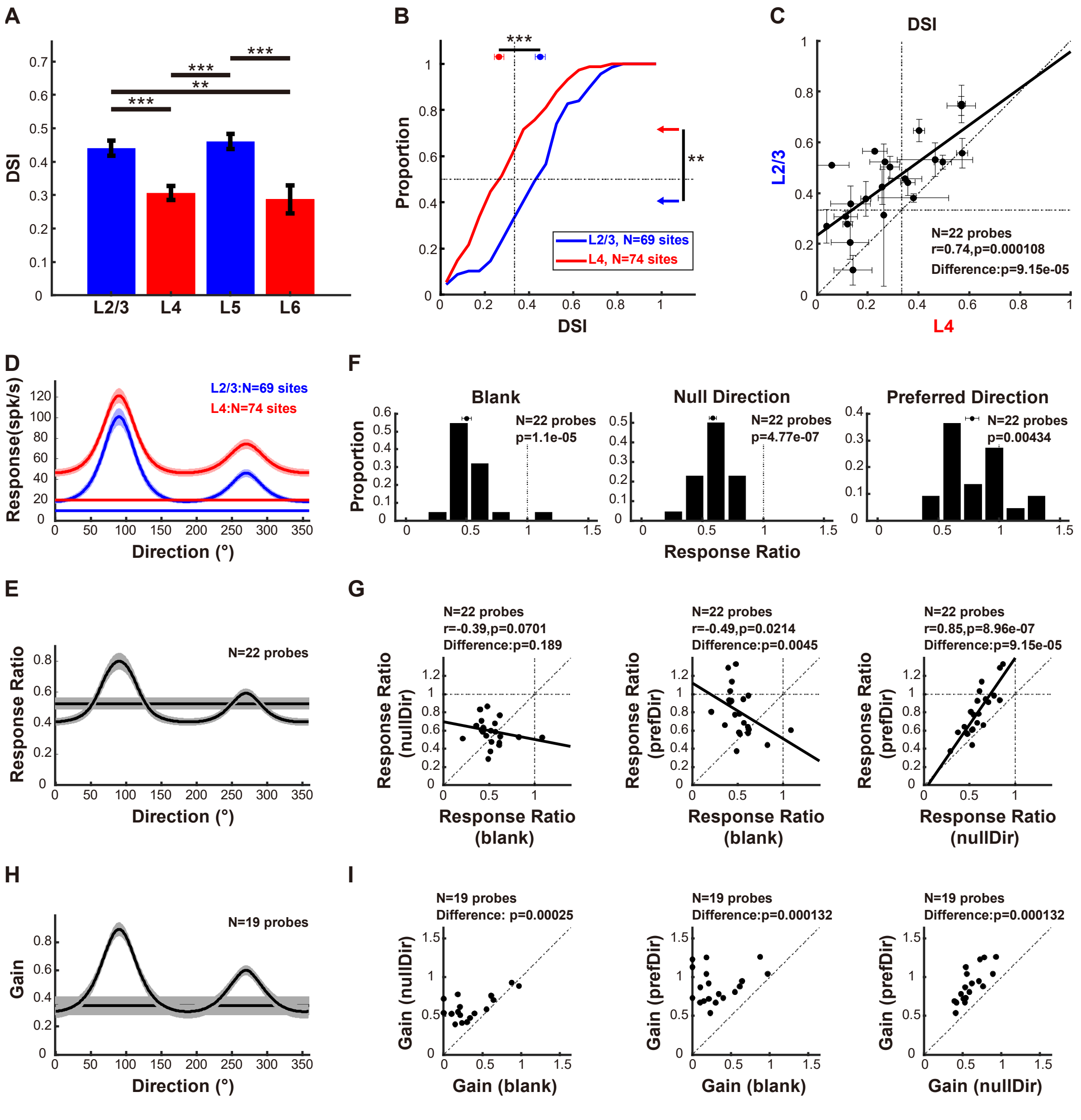

Figure R11. Enhanced direction selectivity in L2/3 and an interlaminar direction-tuned gain from L4 to L2/3.

A. Comparison of DSIs across all layers. The DSIs in L2/3 and L5 were significantly greater than those in L4 and L6.

B. Cumulative distributions of DSIs in L2/3 (blue line) and L4 (red line). L2/3 has stronger direction selectivity than L4.

C. Comparison of DSIs between L2/3 and L4 within the same probe placement. For most of probe placements, L2/3 has stronger direction selectivity than L4.D.

D. Population-averaged direction tuning of response strength within L2/3 (blue lines) and L4 (red lines).

E. Population-averaged direction tuning of the response ratio between L2/3 and L4.

F. Distribution of the response ratio under the indicated stimulus conditions. The vertical dashed line represents a response ratio value of 1.

G. Comparison of the response ratios between the indicated stimulus condition pairs.

H. Population-averaged direction tuning of linear gains in Model II when considering only the signal transmission from L4 to L2/3. The horizontal line represents the linear gain under the blank stimulus.

I. Comparison of linear gains between the indicated stimulus condition pairs.

Other minor comments

-Line 197. 'We observed that for most neuronal pairs within a cortical column, the preferred direction remained largely unchanged, irrespective of whether the neurons were within the same layer or across different layers'. They should mention here that this finding is consistent with a columnar organization of direction preference previously demonstrated in carnivores (e.g. imaging in cat: Shmuel and Grinvald, 1996; imaging in ferret: Weliky et al., 1996; multielectrode recordings in cat: Kremkow et al., 2016).

Reply: Thank you for the suggestion. We have revised the manuscript and added the corresponding references at **lines 245-248, page 10.**

-Line 237. 'suggesting that neuronal activity in the output layer is inherently weaker than that in the input layer.' Perhaps, briefly mention the possibility that general anesthesia (propofol) may affect the differences in response strength between layers. If there is some evidence from awake animals that responses are weaker in output than input layers, they can use this evidence to argue that anesthesia is unlikely to affect the response differences across layers.

Reply: Thank you for the suggestion. Stronger spontaneous activity in the input layers compared to the output layers has been observed in both anesthetized animals (e.g., anesthetized marmosets, Alessandra Angelucci lab, personal communication) and awake animals (primarily in awake monkeys, studies from our lab (Wang et al., 2024) and others (Gur et al., 2005; Poggio et al., 1977; Snodderly & Gur, 1995)).

The effects of propofol on neuronal responses along the cortical column in V1 remain unclear. In one cat study, propofol was found to reduce spontaneous activity in A18/A19, the thalamus, and the reticular formation, though no laminar-specific data were provided (Andrada et al., 2012). Additionally, findings in monkeys have been inconsistent. One study reported reduced spike activity only in deep layers (Redinbaugh et al., 2020), while another found a decrease across all layers, with stronger reductions in superficial layers (Bastos et al., 2021).

To minimize the anesthetic effects of propofol, we carefully monitored the animals' EEG and adjusted the dose to maintain light anesthesia during the experiment. Therefore, we believe that anesthesia is unlikely to have significantly affected the observed differences in spontaneous activities across layers.

We have changed the sentence to "We found that, under blank stimuli, the response strength was

also reduced in the output layers (Figure 4A), which is in line with the reduced spontaneous activity in the output layers of awake macaques (Gur et al., 2005; Poggio et al., 1977; Snodderly & Gur, 1995; T. Wang et al., 2024) and suggests that neuronal activity in the output layers is inherently weaker than that in the input layers." at **lines 285-289, page 11** in the revised manuscript. In the Discussion section, we also added a sentence "Second, the impact of propofol, the anesthetic used in the present study, on neuronal responses throughout the cortical column is a matter of ongoing debate, and it remains unclear whether the differences in response strengths between layers are affected" on **lines 683-687, page 22**.

-Line 275. 'the preservation of neuronal responses in the preferred direction in the output layer suggests that there is selective amplification of the neuronal response from the input layer to the output layer in the preferred direction.' An alternative interpretation is that the output layers suppress responses to all directions (untuned intracortical inhibition) while selectively increasing the response to the preferred direction (tuned intracortical excitation). This mechanism shares certain resemblance with the effects of alertness on directional selective cells in visual thalamus (see Figures 5 and 6 in Hei et al., 2014 from Swadlow's lab).

Reply: Thank you for the suggestion. We have revised the sentence to "However, neuronal responses in the preferred direction are selectively preserved from the input layers to the output layers" **(lines 333-335, page 13)** to focus solely on the experimental findings. We have included the possible interpretations in the later Results section, before introducing the models, at **lines 341-355, page 13** in the revised manuscript.

-Line 439. 'Within a cortical column, we observed that a minority of the neuronal pairs demonstrated a shift in preferred directions of approximately 180 degrees (Figure 2A), corroborating earlier research (Berman et al., 1987).' These direction fractures (180 degrees change in direction preference within neighboring cortical regions) are consistent with a columnar organization of direction preference (see Shmuel and Grinvald, 1996; Weliky et al., 1996; Kremkow et al., 2016). Notice that the existence of this rapid 180 degrees shift in direction preference would be expected if the multielectrode probe is located at the border of two direction-preference columns. Similar rapid shifts have been observed with multielectrode probes inserted tangentially within cat visual cortex (Kremkow et al., 2016, figure 4g-h). Moreover, some neurons at the border of direction columns also have opposite direction preferences for the two eyes (e.g. Olianezhad et al., 2024).

Reply: Thank you for the suggestion. We have now added a sentence "The abrupt shift in preferred directions can be partially explained by the presence of direction fractures within the direction map (Kremkow et al., 2016; Ohki et al., 2005; Shmuel & Grinvald, 1996; N. Swindale et al., 1987) and the observation that some neurons exhibit opposite preferred directions for the two eyes (Olianezhad et al., 2024) in cat V1." at **lines 539-543, page 19** in the revised manuscript.

-Line 461. 'Like the neural circuits responsible for the generation of direction selectivity (Mauss et al., 2017; H. H. Yang & Clandinin, 2018), the direction-tuned gain that we observed could result from increased excitation in the preferred direction (Reichardt, 1961), increased inhibition in the null direction (Barlow & Levick, 1965), or both of these effects.' This interpretation should be

probably mentioned earlier in the paper, when presenting the model in the results section.

Reply: We have revised these sentences to introduce two possibilities for the direction-tuned response ratio between the output and input layers: an untuned gain with a nonlinearity, or a direction-dependent change in the relative strength between excitation and inhibition. These revisions have been moved to **lines 341-355, page 13** of the revised manuscript, before introducing the models.

-Line 467. 'In the V1 of cats (Nishiyama et al., 2019; Ohki et al., 2005; N. Swindale et al., 1987) and ferrets (Li et al., 2006)…, neurons are organized into two-dimensional direction maps'. The references here should include Shmuel and Grinvald, 1996 and Kremkow et al., 2016 for cats, and Weliky et al., 1996 for ferrets.

Reply: We have included these references at **lines 512-515, page 18** in the revised manuscript.

- The legend of Figure 1 does not explain whether the illustrated dot in panel F represents the mean or median.

Reply: Thank you for your careful reading. We have added the sentence "Data are median ± SEMs." in the legend at **line 172, page 7**, as well as to all other legends where similar inter-laminar comparisons are made.

- Figures 3A-B and 4 show three curves for each color without explaining whether they represent mean ± SD in the legend. It would be clearer to use a single curve with a shaded area to illustrate mean ± SD.

Reply: Thank you for pointing out our oversight. These curves represent the mean ± SEMs, and we have added corresponding explanations in the figure legends throughout the manuscript. Additionally, we have modified all related sub-figures across the manuscript to display a single curve with a shaded area for clearer illustration.

- Figure 6 would facilitate the comparison of the three panels (A, B, and C) if the color bars have the same range.

Reply: Thank you for the suggestion. We have updated all three panels to use the same color bar at **lines 471-481, page 17** in the revised manuscript.

Reference

Almasi, A., Sun, S. H., Yunzab, M., Jung, Y. J., Meffin, H., & Ibbotson, M. R. (2022). How Stimulus Statistics Affect the Receptive Fields of Cells in Primary Visual Cortex. *Journal of Neuroscience*, *42*(26), 5198–5211. https://doi.org/10.1523/JNEUROSCI.0664-21.2022

An, X., Gong, H., McLoughlin, N., Yang, Y., & Wang, W. (2014). The mechanism for processing random-dot motion at various speeds in early visual cortices. *PLoS ONE*, *9*(3). https://doi.org/10.1371/journal.pone.0093115

Andrada, J., Livingston, P., Lee, B. J., & Antognini, J. (2012). Propofol and Etomidate Depress Cortical, Thalamic, and Reticular Formation Neurons During Anesthetic-Induced Unconsciousness. *Anesthesia & Analgesia*, *114*(3), 661–669. https://doi.org/10.1213/ANE.0b013e3182405228

Azouz, R., Gray, C. M., Nowak, L. G., & McCormick, D. A. (1997). Physiological properties of inhibitory interneurons in cat striate cortex. *Cerebral Cortex*, *7*(6), 534–545. https://doi.org/10.1093/cercor/7.6.534

Bastos, A. M., Donoghue, J. A., Brincat, S. L., Mahnke, M., Yanar, J., Correa, J., Waite, A. S., Lundqvist, M., Roy, J., Brown, E. N., & Miller, E. K. (2021). Neural effects of propofol-induced unconsciousness and its reversal using thalamic stimulation. *ELife*, *10*, 1–28. https://doi.org/10.7554/ELIFE.60824

Benucci, A., Frazor, R. A., & Carandini, M. (2007). Standing Waves and Traveling Waves Distinguish Two Circuits in Visual Cortex. *Neuron*, *55*(1), 103–117. https://doi.org/10.1016/j.neuron.2007.06.017

Chang, J. T., & Fitzpatrick, D. (2022). Development of visual response selectivity in cortical GABAergic interneurons. *Nature Communications*, *13*(1), 3791. https://doi.org/10.1038/s41467-022-31284-6

Finn, I. M., & Ferster, D. (2007). Computational diversity in complex cells of cat primary visual cortex. *Journal of Neuroscience*, *27*(36), 9638–9647. https://doi.org/10.1523/JNEUROSCI.2119-07.2007

Gentet, L. J., Avermann, M., Matyas, F., Staiger, J. F., & Petersen, C. C. H. (2010). Membrane Potential Dynamics of GABAergic Neurons in the Barrel Cortex of Behaving Mice. *Neuron*, *65*(3), 422–435. https://doi.org/10.1016/j.neuron.2010.01.006

Gray, C. M., & McCormick, D. A. (1996). Chattering cells: Superficial pyramidal neurons contributing to the generation of synchronous oscillations in the visual cortex. *Science*, *274*(5284), 109–113. https://doi.org/10.1126/science.274.5284.109

Gur, M., Kagan, I., & Snodderly, D. M. (2005). Orientation and direction selectivity of neurons in V1 of alert monkeys: Functional relationships and laminar distributions. *Cerebral Cortex*, *15*(8), 1207–1221. https://doi.org/10.1093/cercor/bhi003

Hawken, M. J., Shapley, R. M., Disney, A. A., Garcia-Marin, V., Henrie, A., Henry, C. A., Johnson, E. N., Joshi, S., Kelly, J. G., Ringach, D. L., & Xing, D. (2020). Functional Clusters of Neurons in Layer 6 of Macaque V1. *The Journal of Neuroscience*, *40*(12), 2445–2457. https://doi.org/10.1523/JNEUROSCI.1394-19.2020

Karube, F., & Kisvárday, Z. F. (2011). Axon topography of layer IV spiny cells to orientation map in the cat primary visual cortex (Area 18). *Cerebral Cortex*, *21*(6), 1443–1458. https://doi.org/10.1093/cercor/bhq232

Kim, T., & Freeman, R. D. (2016). Direction selectivity of neurons in the visual cortex is non-linear

and lamina-dependent. *European Journal of Neuroscience*, *43*(10), 1389–1399. https://doi.org/10.1111/ejn.13223

Lee, S. H., Kwan, A. C., Zhang, S., Phoumthipphavong, V., Flannery, J. G., Masmanidis, S. C., Taniguchi, H., Huang, Z. J., Zhang, F., Boyden, E. S., Deisseroth, K., & Dan, Y. (2012). Activation of specific interneurons improves V1 feature selectivity and visual perception. *Nature*, *488*(7411), 379–383. https://doi.org/10.1038/nature11312

Li, Y., Liu, B., Chou, X., Zhang, L. I., & Tao, H. W. (2015). Strengthening of Direction Selectivity by Broadly Tuned and Spatiotemporally Slightly Offset Inhibition in Mouse Visual Cortex. *Cerebral Cortex*, *25*(9), 2466–2477. https://doi.org/10.1093/cercor/bhu049

Lochmann, T., Blanche, T. J., & Butts, D. A. (2013). Construction of Direction Selectivity through Local Energy Computations in Primary Visual Cortex. *PLoS ONE*, *8*(3). https://doi.org/10.1371/journal.pone.0058666

Martinez, L. M., Alonso, J. M., Reid, R. C., & Hirsch, J. A. (2002). Laminar processing of stimulus orientation in cat visual cortex. *Journal of Physiology*, *540*(1), 321–333. https://doi.org/10.1113/jphysiol.2001.012776

Monier, C., Chavane, F., Baudot, P., Graham, L. J., & Frégnac, Y. (2003). Orientation and Direction Selectivity of Synaptic Inputs in Visual Cortical Neurons. *Neuron*, *37*(4), 663–680. https://doi.org/10.1016/s0896-6273(03)00064-3

Nishiyama, M., Matsui, T., Murakami, T., Hagihara, K. M., & Ohki, K. (2019). Cell-Type-Specific Thalamocortical Inputs Constrain Direction Map Formation in Visual Cortex. *Cell Reports*, *26*(5), 1082-1088.e3. https://doi.org/10.1016/j.celrep.2019.01.008

Nowak, L. G., Sanchez-Vives, M. V., & McCormick, D. A. (2008). Lack of orientation and direction selectivity in a subgroup of fast-spiking inhibitory interneurons: Cellular and synaptic mechanisms and comparison with other electrophysiological cell types. *Cerebral Cortex*, *18*(5), 1058–1078. https://doi.org/10.1093/cercor/bhm137

Onat, S., Nortmann, N., Rekauzke, S., König, P., & Jancke, D. (2011). Independent encoding of grating motion across stationary feature maps in primary visual cortex visualized with voltage-sensitive dye imaging. *NeuroImage*, *55*(4), 1763–1770. https://doi.org/10.1016/j.neuroimage.2011.01.004

Onorato, I., Neuenschwander, S., Hoy, J., Lima, B., Rocha, K. S., Broggini, A. C., Uran, C., Spyropoulos, G., Klon-Lipok, J., Womelsdorf, T., Fries, P., Niell, C., Singer, W., & Vinck, M. (2020). A Distinct Class of Bursting Neurons with Strong Gamma Synchronization and Stimulus Selectivity in Monkey V1. *Neuron*, *105*(1), 180-197.e5. https://doi.org/10.1016/j.neuron.2019.09.039

Payne, B. R., & Peters, A. (2002). The Concept of Cat Primary Visual Cortex. In B. R. Payne & A. Peters (Eds.), *The Cat Primary Visual Cortex* (pp. 1–129). Elsevier. https://doi.org/10.1016/B978-012552104-8/50002-X

Poggio, G. F., Doty, R. W., & Talbot, W. H. (1977). Foveal striate cortex of behaving monkey: single-neuron responses to square-wave gratings during fixation of gaze. *Journal of Neurophysiology*, *40*(6), 1369–1391. https://doi.org/10.1152/jn.1977.40.6.1369

Priebe, N. J. (2016). Mechanisms of Orientation Selectivity in the Primary Visual Cortex. *Annual Review of Vision Science*, *2*, 85–107. https://doi.org/10.1146/annurev-vision-111815-114456

Priebe, N. J., & Ferster, D. (2005). Direction selectivity of excitation and inhibition in simple cells of the cat primary visual cortex. *Neuron*, *45*(1), 133–145. https://doi.org/10.1016/j.neuron.2004.12.024

Priebe, N. J., & Ferster, D. (2008). Inhibition, Spike Threshold, and Stimulus Selectivity in Primary Visual Cortex. *Neuron*, *57*(4), 482–497. https://doi.org/10.1016/j.neuron.2008.02.005

Redinbaugh, M. J., Phillips, J. M., Kambi, N. A., Mohanta, S., Andryk, S., Dooley, G. L., Afrasiabi, M., Raz, A., & Saalmann, Y. B. (2020). Thalamus Modulates Consciousness via Layer-Specific Control of Cortex. *Neuron*, *106*(1), 66-75.e12. https://doi.org/10.1016/j.neuron.2020.01.005

Ringach, D. L., Shapley, R. M., & Hawken, M. J. (2002). Orientation selectivity in macaque V1: Diversity and laminar dependence. *Journal of Neuroscience*, *22*(13), 5639–5651. https://doi.org/10.1523/jneurosci.22-13-05639.2002

Rust, N. C., Schwartz, O., Movshon, J. A., & Simoncelli, E. P. (2005). Spatiotemporal elements of macaque V1 receptive fields. *Neuron*, *46*(6), 945–956. https://doi.org/10.1016/j.neuron.2005.05.021

Snodderly, D. M., & Gur, M. (1995). Organization of striate cortex of alert, trained monkeys (Macaca fascicularis): Ongoing activity, stimulus selectivity, and widths of receptive field activating regions. *Journal of Neurophysiology*, *74*(5), 2100–2125. https://doi.org/10.1152/jn.1995.74.5.2100

Sun, S. H., Almasi, A., Yunzab, M., Zehra, S., Hicks, D. G., Kameneva, T., Ibbotson, M. R., & Meffin, H. (2021). Analysis of extracellular spike waveforms and associated receptive fields of neurons in cat primary visual cortex. *Journal of Physiology*, *599*(8), 2211–2238. https://doi.org/10.1113/JP280844

Vigneswaran, G., Kraskov, A., & Lemon, R. N. (2011). Large identified pyramidal cells in macaque motor and premotor cortex exhibit “Thin Spikes”: Implications for cell type classification. *Journal of Neuroscience*, *31*(40), 14235–14242. https://doi.org/10.1523/JNEUROSCI.3142-11.2011

Wang, T., Dai, W., Wu, Y., Li, Y., Yang, Y., Zhang, Y., Zhou, T., Sun, X., Wang, G., Li, L., Dou, F., & Xing, D. (2024). Nonuniform and pathway-specific laminar processing of spatial frequencies in the primary visual cortex of primates. *Nature Communications*, *15*(1), 4005. https://doi.org/10.1038/s41467-024-48379-x

Wang, T., Li, Y., Yang, G., Dai, W., Yang, Y., Han, C., Wang, X., Zhang, Y., & Xing, D. (2020). Laminar Subnetworks of Response Suppression in Macaque Primary Visual Cortex. *The Journal of Neuroscience*, *40*(39), 7436–7450. https://doi.org/10.1523/JNEUROSCI.1129-20.2020

Xing, D., Ringach, D. L., Hawken, M. J., & Shapley, R. M. (2011). Untuned suppression makes a major contribution to the enhancement of orientation selectivity in macaque V1. *Journal of Neuroscience*, *31*(44), 15972–15982. https://doi.org/10.1523/JNEUROSCI.2245-11.2011

Znamenskiy, P., Kim, M. H., Muir, D. R., Iacaruso, M. F., Hofer, S. B., & Mrsic-Flogel, T. D. (2024). Functional specificity of recurrent inhibition in visual cortex. *Neuron*, *112*(6), 991-1000.e8. https://doi.org/10.1016/j.neuron.2023.12.013
